# Daptomycin for the treatment of major gram-positive infections after cardiac surgery

**DOI:** 10.1186/s13019-016-0519-7

**Published:** 2016-08-04

**Authors:** A. Kornberger, B. Luchting, F. Kur, M. Weis, F. Weis, U. A. Stock, A. Beiras-Fernandez

**Affiliations:** 1 Department of Thoracic and Cardiovascular Surgery, University Hospital Goethe University Frankfurt, Theodor Stern Kai 7, Frankfurt, 60590 Germany; 2 Department of Anesthesiology, Frankfurt, Germany; 3 Department of Cardiac Surgery, University Hospital Grosshadern, LM-University, Munich, Germany; 4 Department of Anesthesiology, Clinic of Fuerstenfeldbruck, Fuerstenfeldbruck, Germany

**Keywords:** Daptomycin, Major infection after cardiac surgery, Gram-positive infection, Multi-resistant pathogens

## Abstract

**Background:**

Infection is a main cause of morbidity and mortality after heart surgery, with multi-resistant pathogens increasingly representing a challenge. Daptomycin provides bactericidal activity against gram-positive organisms that are resistant to standard treatment including vancomycin.

**Methods:**

A cohort of cardiac surgical patients, treated with daptomycin for major infection at two tertiary care centers, were retrospectively studied with a particular focus on the type of infection, causative pathogens and co-infections, daptomycin dosage, adverse events and outcome in order to provide evidence for the efficiency and safety of daptomycin in a distinct high-risk patient population.

**Results:**

Sixty-five patients (87.7 % males, 60.4 ± 13.5 years) who had undergone aortic surgery (20.0 %), ventricular assist device (VAD) implantation (21.5 %), combined procedures (21.5 %), coronary artery bypass grafting (12.3 %), isolated valve surgery (15.4 %) and heart transplantation (7.7 %) were diagnosed with catheter-related infection (26.1 %), valve endocarditis (18.8 %), sternal wound (13.0 %), VAD-associated (11.6 %), cardiac implantable electrophysiological device (CIED)-associated (4.1 %), respiratory tract (4.3 %), bloodstream (4.3 %) and other infection (4.3 %). In 13.0 %, no focus of infection was identified though symptoms of severe infection were present. The most frequent pathogens were *Staphylococcus epidermidis* (30.4 %), *Staphylococcus aureus* (23.1 %) and *Enterococcus* species (10.1 %). Daptomycin doses ranging from 3 mg/kg every 48 h to 10 mg/kg every 24 h were administered for 15.4 ± 11.8 days. 87.0 % of the cases were classified as success, 7.2 % as treatment failure and 5.8 as non-evaluable. Adverse events were limited to one case of mild and one case of moderate neutropenia with recovery upon termination of treatment.

**Conclusion:**

Daptomycin proved safe and effective in major infection in high-risk cardiac surgical patients.

## Background

Major infection is a severe complication in cardiac surgical patients and may consist of sternal wound infection, bloodstream or catheter-related infection, native or prosthetic valve endocarditis, or infection associated with other implantable devices such as CIEDs, VADs or vascular prosthetic material. Recent investigations of large patient cohorts showed incidences of major infection after cardiac surgery amounting to up to nearly 5 % [1–8]. Investigation into the microbiological features of major infections in cardiac surgical patients showed that the majority of isolates are gram-positive [2, 7, 9–13]. As the number of gram-positive bacteria, resistant to standard treatment (aminopenicillins or cephalosporines) is substantial, the initial choice of drug is of utmost importance [14–16].

Vancomycin, the first-choice drug used against methicillin-resistant gram-positive pathogens, is associated with considerable side effects. Of these, nephrotoxicity is particularly unfavorable in the cardiac surgical population in whom acute kidney injury was reported to occur at a rate of up to 30 % and subtle renal injury is present in virtually all patients in the early postoperative phase [17–19]. Additionally, vancomycin does not achieve high tissue levels and has limited activity in the biofilm layer on implanted materials. Correct dosage requires drug monitoring and continuous application, which is currently favored, is not suitable for general wards [20–22]. Therefore, the introduction of alternative substances such as linezolid, tigecyclin, quinupristin-dalfopristin and daptomycin has met with particular interest [23].

Daptomycin is a cyclic lipopeptide derived from *Streptomyces roseosporus* as a fermentation product and has rapid concentration-dependent bactericidal activity against most gram-positive pathogens including methicillin-resistant *Staphylococcus aureus* (MRSA), vancomycin intermediate susceptible *Staphylococcus aureus* and vancomycin-resistant enterococci [24–28]. In 2003, the U.S. Food and Drug Administration (FDA) approved daptomycin for the treatment of complicated skin and soft tissue infections, and in 2006, an additional FDA approval was granted for the treatment of bloodstream infections and right-sided endocarditis. In Europe, Daptomycin was approved for the treatment of complicated skin and soft tissue infections in 2006, and for the treatment of *Staphylococcus aureus* right-sided infective endocarditis and bacteremia in 2007 [29–31]. Among the anti-infective substances recently introduced, daptomycin stands out for its excellent tissue penetration, killing rate and penetration into biofilm layers.

The aim of the present study is to review our clinical experience with daptomycin for the treatment of major gram-positive infections after heart surgery with a focus on safety, tolerability and efficacy, thus providing complementary insight into the application of daptomycin in challenging infections in a highly specific patient cohort.

## Methods

### Patients, definitions and data collection

We retrospectively identified all consecutive adult cardiac surgical patients with major infective complications after cardiac surgery who were treated with daptomycin at two tertiary centers from 2008 to 2009 and from 2011 to 2014, respectively. None of the patients were treated with daptomycin in the setting of a clinical trial. The patients at the first center provided informed consent for retrospective evaluation of their data including reporting to the European Cubicin (Novartis Pharma GmbH, Nuremberg, Germany) Outcomes Registry and Experience (EU-CORE) database. For the part of the study implemented at the second center, institutional review board approval including a waiver of informed consent was granted (AZ 511/15).

Data were obtained by reviewing medical records with a special focus on signs and symptoms of infection, on the one hand, and findings potentially indicating adverse effects of daptomycin, on the other. Diagnosis of infection relied on clinical assessment, imaging, laboratory parameters and microbiological findings. Therapy with daptomycin was instituted at the discretion of the senior physicians in charge of the respective intensive care unit (ICU) and independently of the present study, which is a purely retrospective analysis of daptomycin use that was not initiated until after completion of treatment in all patients enrolled.

The clinical outcomes upon completion of daptomycin therapy were retrospectively evaluated and classified as “success” where clinical signs and symptoms were found to have resolved and/or cultures returned negative and/or infection markers decreased. Outcomes were classified as “non-evaluable” where the response to therapy with daptomycin could not be determined, for example due to the effects of secondary infection or insufficient data, and as “failure” where the infection treated with daptomycin persisted, worsened or recurred.

### Statistical analysis

Descriptive statistics were calculated using means and standard deviations, frequencies and percentages as appropriate. Data analysis was performed using SPSS 22.0 (SPSS, Inc., Chicago, US).

## Results

Daptomycin was administered in 69 cases of major infection in 65 patients (mean age 60.4 ± 13.5 years, 87.7 % male). Two patients had more than one course of daptomycin treatment, with the subsequent courses not related to the respective initial infection. Demographic parameters and baseline characteristics, as well as diagnostics, treatment and outcomes are summarized in Table 1.

**Table 1:** Summary of baseline, infection, diagnostic, treatment and outcome parameters

Demographic data and comorbidities	(patients, *n* = 65)	Cardiac surgical procedure	(patients, *n* = 65)
Age [years]	Mean 60.44 ± 13,51	Aortic surgery	13 (20.0 %)
Male	57 (87.7 %)	VAD implantation	14 (21.5 %)
Weight [kg]	Mean 80.57 ± 14.64	Combined procedures	14 (21.5 %)
Diabetes	17 (26.2 %)	CABG	8 (12.3 %)
COPD	6 (9.2 %)	Isolated valve surgery	10 (15.4 %)
Rheumatic disease	1 (1.5 %)	Heart transplantation	5 (7,7 %)
Hemodialysis	9 (13.8 %)	CABG + CIED removal	1 (1.5 %)
Classified as multi-morbid	31(47.7 %)	Classified as high-risk procedure	32 (67.7 %)
Focus of infection	(courses of treatment, *n* = 69)	Pathogens isolated	(courses of treatment, *n* = 69)
Central venous catheter-associated infection	16 (23.2 %)	Staphylococcus epidermidis	21 (30.4 %)
Valve endocarditis	13 (18.8 %)	Staphylococcus aureus, MRSA	9 (13.0 %)
Sternal wound infection	9 (13.0 %)	Staphylococcus aureus, MSSA	7 (10.1 %)
VAD-associated infection	8 (11.6 %)	Enterococcus spp.	7 (10.1 %)
CIED-associated infection	3 (4.3 %)	Other	6 (8.7 %)
Respiratory tract infection	3 (4.3 %)	No causative organism identified	19 (27.5 %)
Bloodstream infection	3 (4.3 %)		
Infection of arterial lines	2 (2.9 %)		
Other infection	3 (4.3 %)		
No focus of infection identified	9 (13.0 %)		
Secondary infection	(courses of treatment, *n* = 69)	Diagnostics	(courses of treatment, *n* = 69)
Present in	34 (49.3 %)	Clinical signs and symptoms only	15 (21.7 %)
Fungal pathogen	24 (70.6 %)	Positive swab/tissue sample from infection site	23 (33.3 %)
Gram-negative rods	6 (17.6 %)	Positive blood culture	15 (21.7 %)
Staph. aureus	2 (5.9 %)	Echocardiographic findings	13 (18.8 %)
Coagulase-negative staphylococcus	1 (2.9 %)	CT findings	4 (5.8 %)
Propionibacterium acnes	1 (9.1 %)	Positive culture from catheter tip	15 (21.7 %)
		Multiple diagnostics	10 (14.5 %)
Surgical/interventional measures	(courses of treatment, *n* = 69)	Classification of outcome	(courses of treatment, *n* = 69)
Valve replacement for endocarditis	12 (17.4 %)	Success	60 (87.0 %)
Wound debridement (with/without removal of foreign material, placement of drains, vacuum assisted closure)	17 (24.6 %)	Failure	5 (7.2 %)
Removal of infected venous or arterial catheters	19 (27.5 %)	Non-evaluable	4 (5.8 %)
Placement of drains	2 (2.9 %)		

In 79.7 % of the cases, patients had received antibacterial treatment with other substances including penicillins, cephalosporins, carbapenems, aminoglycosides, glycopeptides and oxazolidinones before the first dose of daptomycin. In 5 cases of endocarditis, daptomycin was initiated before surgery. In all other cases, daptomycin was administered after cardiac surgical procedures.

The study cohort comprised 13 (20.0 %) patients who had undergone aortic surgery including isolated aortic valve replacement as well as aortic replacement procedures. 14 (21.5 %) patients each had undergone VAD implantation and combined procedures. CABG had been performed in 8 (12.3 %) further patients, isolated valve surgery in 10 (15.4 %) and heart transplantation in 5 (7.7 %). A total of 32 (67.7 %) were classified as high-risk procedures.

Investigation of the initial focus of infection showed that central venous catheter-associated infection (*n* = 16, 23.2 %) was the most frequent indication for treatment with daptomycin, followed by valve endocarditis (*n* = 13, 18.8 %), sternal wound infection (*n* = 9, 13.0 %) and VAD-associated infection (*n* = 8, 11.6 %). CIED-associated, respiratory tract and bloodstream infection accounted for 3 (4.3 %) cases each, 2 (2.9 %) patients were diagnosed with infection of arterial lines, and 3 (4.3 %) with other infection. No focus of infection was identified in 9 (13.0 %) cases.

*Staphylococcus epidermidis* (*n* = 21, 30.4 %) was the most frequent pathogen isolated, followed by methicillin resistant (*n* = 9, 13.0 %) and methicillin-susceptible (*n* = 7, 10.1 %) *Staphylococcus aureus* and *Enterococcus* species (*n* = 7, 10.1 %). No causative organism was identified in 19 (27.5 %) cases. Secondary infection was present in 34 (49.3 %) cases, with fungal pathogens accounting for 70.6 % (*n* = 24) of the secondary infections. The remaining secondary infections were caused by gram-negative rods (*n* = 6, 17.6 %), *Staphylococcus aureus* (*n* = 2, 5.9 %), coagulase-negative *Staphylococcus* species (*n* = 1, 2.9 %) and *Propionibacterium acnes* (*n* = 1, 9.1 %).

In a third of the cases (*n* = 23, 33.3 %), a causative organism was identified by microbiological examination of swabs or tissues from the infection sites. Blood cultures and cultures from catheter tips were positive in 15 (21.7 %) cases each. Echocardiographic and CT findings supported the diagnosis of major infection in 13 (18.8 %) and 4 (5.8 %) of the cases, and 15 (21.7 %) cases were diagnosed on the basis of clinical signs and symptoms only. In 10 (21.7 %) cases, the diagnosis of major infection was established using more than one diagnostic modality.

Treatment with daptomycin lasted 15.4 ± 11.8 days (range 2–92 days). In all but 12 (17.4 %) cases, patients received daptomycin once daily. Doses ranged from 3 mg/kg at intervals of 48 h to 10 mg/kg at intervals of 24 h. The median dose was 6 mg/kg/24 h and administered in 20 (29.05) cases, while doses exceeding 6 mg/kg/day were administered in 15 (21.7 %) cases. Patients with sternal wound infection received between 5 mg/kg and 8 mg/kg at intervals of 24 h, those with catheter-related infection from 3 mg/kg at intervals of 48 h to 7 mg/kg at intervals of 24 h, those with VAD-associated infection between 3 mg/kg and 7 mg/kg per day, and those with endocarditis up to 10 mg/kg per day.

The courses of daptomycin for VAD-associated infection, of which three were administered for mediastinitis with VAD involvement, two for deep driveline infection, and one each for abdominal wall abscess with driveline involvement, thoracic wall abscess with VAD involvement and intrapericardial hematoma and intrathoracic abscesses, showed considerable variation and consisted of between 3 mg and 7 mg/kg per day.

Interventional measures or surgical therapy supplementing the antimicrobial regimen were performed in 50 (72.5 %) of the cases. Removal of infected catheters (*n* = 19, 27.5 %) was the most frequent supplementary measure, followed by wound debridement (*n* = 17, 24.6 %) with or without removal of foreign material, placement of drains and/or vacuum-assisted closure. Valve replacement for endocarditis was performed in 12 (17.4 %) cases, and drains were placed in 2 (2.9 %).

The vast majority of cases (*n* = 60, 87.0 %) were classified as success. 4 (5.8 %) were classified as “non-evaluable” because improvement could not be assessed due to secondary infection, severe deterioration and death for other causes, or insufficient data. 5 (7.2 %) cases were classified as “failure” because infection persisted or kept returning.

Serum creatinine was determined once or twice daily in the course of routine ICU panels. The baseline creatinine clearance, defined as the level before the first dose of daptomycin, was 54.3 ± 38.7 ml/min. Upon completion of treatment with daptomycin, a creatinine clearance of 53.7 ± 42.5 ml/min was measured. A significant deterioration of creatinine clearance and need for renal replacement therapy during daptomycin treatment was noted only in a patient who received 3 courses of daptomycin for deep sternal wound infection with VAD involvement at intervals of > 1 month and experienced multi-organ failure secondary to sepsis and heart failure.

Creatinine phosphokinase (CPK) levels were monitored closely at both centers. Increases occurred in nearly all patients, but as fluctuations were found to be associated with cardiac surgery, no valid conclusion could be drawn from the evaluation of CPK values at baseline, during and after daptomycin. Monitoring of white blood cell (WBC) levels for leukopenia yielded minimum values between 1.9 G/l and 19.4 G/l. A drop in WBC to values below the normal range was noted only in two patients of whom one showed only temporary mild leukopenia with a minimum WBC count of 3.46 G/L. In the second, a decrease from 12.6 G/L to 1.72 G/L during daptomycin treatment with a recovery to 3.35 G/L upon termination of treatment was noted. Beyond this, no adverse events or undesired side effects attributable to daptomycin treatment were registered.

## Discussion

The outcomes of our previous work, which demonstrated the efficacy of daptomycin in cardiac surgical patients [32], are confirmed by the results of the present study. With 87.0 % of the cases assessed as clinical success and 7.2 % as treatment failure, our current results are comparable to the success and failure rates of 79 and 7.5 % reported by the European Cubicin Outcome Registry and Experience EU-CORE [33]. Our cohort, however, was highly specific in that our patients had undergone a variety of mostly high-risk cardiac surgical procedures that resulted in considerable surgical trauma and the effects of cardiopulmonary bypass adding to a post-surgical risk profile that resulted not only from the underlying cardiac conditions, but also from comorbidities, frailty, presence of foreign material including heart valves and VADs and, in the case of heart transplantation, need for immunosuppression. In addition, a significant share of our patients were treated for challenging conditions such as deep sternal wound infection, CIED- or VAD-associated infection and endocarditis, while skin and soft tissue infections made up 44 % of the infections registered in the EU-CORE database, with smaller numbers treated for bacteraemia (22 %), endocarditis (12 %) or osteomyelitis (6 %).

In our patient cohort, endocarditis, sternal wound infection and VAD-associated infection were the most frequent types of infection specific to cardiac surgical patients. Of these, sternal wound infection has variously been found to be a promising area of application for daptomycin, which was reported to be safe and effective [32, 34] and to have yielded favourable mid-term results [35]. With regard to osteomyelitis not limited to the sternum, encouraging results for the application of daptomycin were published in a number of single-center reports and reviews [36, 37], as well as from the CORE and EU-CORE registries. In the EU-CORE database, 220 of 3621 patients registered from 2006 to 2010 were treated for osteomyelitis, with the most common sites of infection being knees and hips, and the most common pathogens *Staphylococcus aureus* and coagulase-negative staphylococci. Cure and improval were reported in 23 % and 52 % of patients treated for osteomyelitis, respectively, yielding an overall clinical success rate of 75 %. Notably, success was similar between MRSA and MSSA and lowest treatment failure rates were observed for coagulase-negative staphylococcal pathogens [38]. From the US-based CORE registry, improval rates ≥ 90 % were reported for patients treated with daptomycin for osteomyelitis, with a higher success rate in in-patients vs. out-patients [39] and a trend towards higher improval rates in patients receiving a daily dose of ≥ 6 mg/kg [40].

The issue of higher dosage in patients with particularly severe infection, including osteomyelitis, was repeatedly addressed, and it was pointed out that daptomycin was well-tolerated at doses ≥ 6 mg per kg to which better outcomes were attributed [37, 40–43]. The Infectious Diseases Society of America, in its 2011 guidelines [44], not only included daptomycin, though not licensed for the treatment of osteomyelitis, as an option for the treatment of MRSA osteomyelitis, but in keeping with the assumption that higher daily dosages will compensate for low vascularisation of bone tissue, recommended the maximum approved daily dose of 6 mg/kg daptomycin. Considering the data published to date, dosage and the possibility to apply high-dose daptomycin regimens may be assumed to be of special importance in the context of osteomyelitis even though evaluation of the EU-CORE database showed no dose-related outcomes in patients treated for osteomyelitis [38].

When it comes to justifying administration of high-dose daptomycin, cardiac surgical patients with sternal wound infections represent a distinct subgroup in that sternal osteomyelitis may rapidly progress to frank and potentially lethal mediastinitis. Additionally, the issues of bone penetration of antibiotics and vascularisation of osseous structures must be viewed against the background of the fact that the sternal blood supply is impaired due to sternotomy and sternal closure and, in the case of CABG, unilateral or bilateral internal thoracic artery harvesting. Nevertheless, data guiding daptomycin dosage in cases of sternal osteomyelitis and mediastinitis are scarce. In our patients, those treated for sternal wound infection received daptomycin at daily doses between 4 and 8 mg/kg, thus contrasting with previous authors who reported administration of a standard daily dose of 6 mg/kg [45].

A role for high-dose daptomycin was also suggested for the treatment of infective endocarditis (IE) including left-sided and double-sided IE [43, 46–48]. Of the patients registered in the EU-CORE database from 2006 to 2010, 10 % were treated for IE including 69 % of left-sided IE. The majority (59 %) received 6 mg/day, while doses ≥ 6 and up to 12 mg/day were administered in 26 % of cases, with coagulase-negative staphylococcus and *Enterococcus faecalis* associated with daptomycin dosages exceeding 6 mg/kg [49].

Our patient cohort comprised 13 patients with IE. Contrasting with a share of only 50 % in the EU-CORE database [49], all but one treated for postoperative prosthetic valve endocarditis underwent surgical treatment. This is noteworthy in that evaluation of EU-CORE registry data showed that surgical intervention is typically associated with the most severe forms of IE, on the one hand, and that patients receiving surgical intervention had a higher success rate than those managed medically, on the other [49]. The overall success rate determined for treatment of IE in the framework of the EU CORE registry was 80 % (91 % for right-sided and 76 % for left-sided IE, and higher dosage regimens were found to be associated with a higher success rate of 90 % in patients treated with ≥ 8 mg/day (91 % for right-sided and 89 % for left-sided IE) [49]. A slightly lower success rate of 85.9 %, with success and failure rates similar in the left and right-sided IE groups, was reported from a multi-centric study including 70 patients who received daily doses between 8.2 and 10.0 mg/kg per day [47]. Additionally, successful treatment of IE using daily doses up to 12 mg/kg was reported [46, 50, 51]. This is in keeping with the results of our retrospective analysis, which also showed that the majority of patients treated for endocarditis received ≥ 6 mg and up to 10 mg/kg per day.

In the report from the EU-CORE registry, it was additionally pointed out that the rate of CIED-associated infections and the number of patients requiring device extraction in addition to antimicrobial therapy has risen substantially over the past few years [49]. Though the number of patients with CIED-associated infection in our patient cohort was low, CIED-associated infection represents a potentially fatal complication that requires aggressive treatment. This is reflected by the fact that, in a recent study of 25 patients treated for CIED-associated infection, daily doses between 6.4 and 10.7 mg/kg were administered, with lead extraction performed in 88 % of patients. Treatment success was 80 %, with complete microbiological success observed in 92 % [52]. Of a further collective of nine patients treated for CIED-associated infection with daptomycin, 7 had their CIED removed. Eight patients, including those who did not undergo system removal, were cured with a daily dosage of 6 mg/kg [53]. Further literature on treatment of CIED-associated infection with daptomycin in scarce and mostly limited to individual case reports [54–57] even though daptomycin, with its ability to penetrate into biofilms [58–60], is well suited for the treatment of CIED-associated infections, which are characterized by biofilm development on leads.

This particular aspect is shared by VAD-associated infection, where biofilm development on device surfaces and drivelines also renders the eradication of infection difficult. Our study population comprised > 20 % of VAD recipients, reflecting that antimicrobial treatment using daptomycin was commenced as soon as major infection in a VAD recipients was suspected. The particular severity of VAD-associated infection is reflected by the fact that even though two of our VAD recipients received three courses of daptomycin each, deep driveline infection kept recurring in one, while the other finally succumbed to fungal mediastinitis with VAD involvement. Our retrospective analysis has shown that daptomycin was frequently chosen for the treatment of VAD recipients because of its bactericidal activity against multi-resistant gram-positive organisms, its good penetration into biofilms, and because it has variously proven efficient in treating infection in VAD recipients [61–63].

Of the remaining patients, those in whom the respiratory tract was designated as the primary focus of infection deserve mention because daptomycin is expressly not recommended for treatment of pulmonary infections [64, 65]. In these cases, the respiratory tract was indicated as the initial focus of infection and the microbiological results obtained from the patients’ respiratory secretion served to guide antibiotic management, but daptomycin was not chosen to treat gram-positive respiratory tract infection as such, but to prevent seeding of pathogens from the respiratory tract in severely compromised patients in an ICU setting.

In the largest subgroup of our patient cohort, i.e. patients with catheter-associated infections, we made more liberal use of daptomycin than recommended by the Infectious Diseases Society of America in its 2009 Update of its Clinical Practice Guidelines for the Diagnosis and Management of Intravascular Catheter-Related Infection. These recommend alternative agents such as daptomycin for empirical therapy of catheter-related bloodstream infections in health care settings with an elevated prevalence of MRSA in which the preponderance of MRSA isolates have vancomycin minimum inhibitory concentration values > 2 μg/ml. Additionally, daptomycin is listed as an option in patients with catheter-related bloodstream infection due to ampicillin- and vancomycin-resistant enterococci and patients receiving hemodialysis with catheter-related bloodstream infection due to vancomycin-resistant enterococci [66]. In contrast to the dosages of 6 mg/kg and 6–8 mg/kg per day recommended by these guidelines, our patients with catheter-related infection mostly received daptomycin at lower doses ranging from 3 mg/kg at intervals of 48 h to 7 mg/kg at intervals of 24 h. Treatment success in patients with catheter-related infection was nevertheless achieved in 72.2 % of cases, compared to a total success rate of 83.7 %.

Assessment of the overall outcome in our patient cohort yielded a favourable profile for daptomycin in terms not only of efficacy and clinical success rates, but also with regard to tolerability and safety even in high doses. This applies, in particular, to muscle toxicity and drug-related CPK elevations [33, 67, 68], which were reported to have occurred more frequently in patients receiving daily doses > 6 mg/kg [49], but also to renal impairment [69, 70] and leukopenia [71, 72]. Regular monitoring of our patients for CPK, creatinine clearance and WBC implemented showed no relevant changes in creatine clearance and CPK levels from baseline to completion that were attributed to daptomycin, and only one case of moderate leucopenia that resolved upon termination of treatment.

### Limitations

The shortcomings of the present study mainly consist in its retrospective design, which rendered evaluation of the treatment success difficult in some cases and led to 4 (5.8 %) cases being classified as “non-evaluable” because improvement could not be assessed due to secondary infection, severe deterioration and death for other causes, or insufficient data.

## Conclusions

In conclusion, daptomycin treatment was successful in the clinical management of patients with major infection after cardiac surgery and therefore represents a promising alternative to conventional antimicrobial treatment of challenging infections in cardiac surgical patients. Our two-center experience confirms efficacy, as well as tolerability and non-toxicity, in a highly specific patient population that is characterized by surgical trauma adding to cardiac morbidity, comorbidity and frailty. Reports on higher doses in patients treated for particularly severe infections yielding improved success rates without causing more adverse events warrant further investigation in cardiac surgical patients with major infection, too.

## Abbreviations

CIED, cardiac implantable electrophysiological device; CORE, US-based cubicin Outcomes Registry and Experience; CPK, creatinine phosphokinase; EU-CORE, European Cubicin Outcome Registry and Experience; FDA, Food and Drug Administration; ICU, intensive care unit; IE, infective endocarditis; MRSA, methicillin-resistant *Staphylococcus aureus*; MSSA, methicillin-susceptible *Staphylococcus aureus*; VAD, ventricular assist device; WBC, white blood cell count
